# Intensification of Cr(VI) adsorption using activated carbon adsorbent modified with ammonium persulfate

**DOI:** 10.1038/s41598-024-68105-3

**Published:** 2024-07-23

**Authors:** Fazel Zahakifar, Maryam Dashtinejad, Hamid Sepehrian, Mohammad Samadfam, Javad Fasihi, Ali Yadollahi

**Affiliations:** 1 Nuclear Fuel Cycle Research School, Nuclear Science and Technology Research Institute, P. O. Box 11365/8486, Tehran, Iran; 2 Department of Energy Engineering, Sharif University of Technology, Azadi Ave., Tehran, Iran

**Keywords:** Granular activated carbon, Modification, Ammonium persulfate, Cr(VI), Adsorption, Intensification, Chemical engineering, Environmental chemistry, Chemical synthesis

## Abstract

Granular activated carbon has been modified by ammonium persulfate as a new adsorbent for Cr(VI) adsorption from aqueous solutions. The adsorbent was characterized by nitrogen adsorption–desorption isotherm data and infrared spectroscopy. The impact of different factors, such as the initial pH level of the solution, time, temperature, ionic strength, and initial concentration of the Cr(VI) ion, on the adsorption efficiencies of the adsorbent has been studied by batch experiments. Kinetic studies and the adsorption thermodynamics of Cr(VI) with ammonium persulfate-modified activated carbon adsorbent were carefully studied. The results showed that the Cr(VI) adsorption follows a pseudo-second-order kinetic model and the adsorption reaction is endothermic and spontaneous. The adsorption isotherm was scrutinized, and the fitting results showed that the Langmuir model could well represent the adsorption process. The maximum adsorption capacity of Cr(VI) onto persulfate-modified activated carbon was 108.69 mg g^−1^. The research results showed that using persulfate-modified activated carbon adsorbent can greatly remove Cr(VI) from aqueous solutions.

## Introduction

In many industries, including mining, wood preservation, tanning leather, paint pigments, textile printing and dyeing, refractories, aerospace, and electroplating, chromium (VI) metal is widely employed^1,2^. Because of this metal's extensive use, chromium-containing wastewater is produced.

The trivalent form of Cr is an essential nutrient^3–5^. But the hexavalent form of Cr is toxic, carcinogenic, and mutagenic^1,6^. Therefore, separating Cr from wastewaters is necessary due to its economic and environmental importance.

To treat the effluent containing Cr compounds, several methods have been studied, including chemical reduction to convert Cr(VI) into Cr(III) cations^7^, reverse osmosis^8^, membrane^9^, and adsorption^10,11^. Among these methods, adsorption by adsorbent is a common and widely used method. In past studies, activated carbon (AC) has been introduced as an adsorbent for the removal of Cr(VI) from wastewater^12–18^. However, the use of AC for Cr(VI) adsorption from aqueous solutions is limited due to its low adsorption capacity^18,19^. Therefore, it is necessary to modify its surface to increase the adsorption of Cr(VI)^20^. Important properties of the AC include functional groups attached to the edges of the surface layer, such as phenol, carbonyl, and carboxyl^21^. Chemical methods can be used for functional group modification^21^. The AC has been modified by utilizing different materials such as chitosan, metal oxides, and surfactants^22–24^.

To reflect its novelty, this research investigates the intensification of Cr(VI) adsorption from aqueous solutions using modified AC. To the best of our knowledge, a similar previous study on the kinetics, thermodynamics, and isotherms of Cr(VI) adsorption with ammonium persulfate-modified activated carbon adsorbent has not been reported. The AC was modified with ammonium persulfate to increase the Cr(VI) adsorption capacity significantly. BET, and FTIR characterized the prepared adsorbent. The impact of various parameters such as pH, time, temperature, initial concentration, and ionic strength on the adsorption capacity of Cr(VI) was evaluated. The kinetic, thermodynamic, and isotherm studies of Cr(VI) adsorption with ammonium persulfate-modified activated carbon adsorbent were also carefully scrutinized.

## Materials and methods

### Materials

Nitric acid, hydrochloric acid, sodium hydroxide, sodium chloride, ammonium persulfate, sulfuric acid, potassium dichromate, and granular activated carbon (Cas No 7440-44-0) were purchased from Merck company. Activated carbon had a particle size of 20–60 mesh, a melting point of 4096.15 K and an auto-ignition temperature of 723.15 K.

### Laboratory equipment

Brunauer–Emmett–Teller (BET) nitrogen gas adsorption and desorption were used to determine the adsorbent pores' surface area, volume, and diameter (Quantachrome NOVA 2200E). Fourier transform infrared spectroscopy (FTIR) was utilized to identify the adsorbent functional groups (Bruker, Vector-22 FTIR). A pH meter equipped with a glass electrode (Scott, CG-841) was employed to adjust the pH of the solutions. The mechanical shaker (Gallenkamp IOI 400.XX2.C) was used for adsorption experiments. The concentration of Cr in the solution was assessed by the inductively coupled plasma atomic emission spectroscopy (ICP-AES), VARIAN LIBERTY150AX TURBO model from Australia.

## Preparation of modified activated carbon adsorbent

First, 50 mL of saturated ammonium persulfate solution was prepared in 1 mol L^-1^ sulfuric acid. 5 g of granular activated carbon was added to the prepared solution. The mixture was then mixed at ambient temperature for 24 h with a shaker (Infors AG CH-4103, Bottmingen, Switzerland) at 150 rpm. After this step, the activated carbon was washed several times with distilled water and dried in the oven at 100 °C for 12 h. The modified activated carbon was heated under a nitrogen atmosphere for 5 h at 650 °C.

### Characterization of modified activated carbon adsorbent

The physical and chemical properties of the adsorbent were investigated using nitrogen adsorption and desorption techniques, and Fourier transform infrared spectroscopy.

### Cr(VI) adsorption experiments

The batch method was used in the adsorption studies of Cr ions on modified activated carbon. In this process, 0.03 g of adsorbent with 10 mL of solution (100 mg L^-1^) was placed at 298.15 K for 3 h in a shaker incubator at 150 rpm. After separating the adsorbent from the solution, the amount of soluble Cr was determined by ICP-AES. The distribution coefficient (K_d_), adsorption percentage, and adsorption capacity (q) were calculated by the following Eqs. ^25–29^:

1 $${\text{K}}_{d} = \frac{{{\text{C}}_{i} - {\text{C}}_{f} }}{{{\text{C}}_{f} }} \times \frac{{\left\{ {\text{V}} \right\}}}{{\left\{ {\text{m}} \right\}}}$$

2 $${\text{Adsorption}}\left( \% \right) = \frac{{\left( {{\text{C}}_{i} - {\text{C}}_{f} } \right)}}{{{\text{C}}_{f} }} \times 100$$

3 $${\text{q}} = \left( {{\text{C}}_{i} - {\text{C}}_{f} } \right) \times \frac{{\left\{ {\text{V}} \right\}}}{{\left\{ {\text{M}} \right\}}}$$

where C_i_, C_f_, V, and m are the initial concentration of Cr (mg L^−1^), the equilibrium concentration of Cr(VI) (mg L^−1^), solution volume (mL), and adsorbent mass (g), respectively. To Cr adsorption experimental tests were carried out three times, and the average results were reported.

Also, in the isotherm study, the feed solution in the range of 5–600 mg L^−1^ was used. To investigate the thermodynamics of adsorption, the experiments were carried out at a temperature of 298.15–338.15 K.

To investigate the adsorption kinetics, Cr(VI) adsorption experiments were performed at different times (10, 30, 60, 150, 300, 600, and 1500 min). In this process, 0.03 g of adsorbent with 10 mL of solution (100 mg L^−1^) was placed at 298.15 K in a shaker incubator at 150 rpm.

## Results and discussion

### Nitrogen adsorption and desorption study

**Figure 1:**
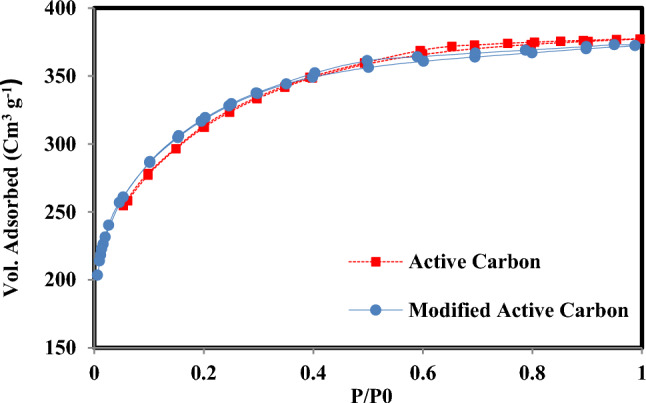
The adsorption and desorption isotherms of nitrogen.

The nitrogen adsorption and desorption isotherms are depicted in Fig. 1. Table 1 showcases the specific surface area, pore volume, and average pore size associated with activated carbon and ammonium persulfate-modified activated carbon.

**Table 1:** Properties of AC and modified AC with ammonium persulfate.

Sample	volume of pores (cc.g^−1^)	Specific surface area (m^2^.g^−1^)	Average size of pores (nm)
AC	0.58	1099	2.12
Modified AC	0.51	974	0.96

The reduction of the average pore size from 2.12 nm to 0.96 nm in modified activated carbon indicates that ammonium persulfate particles were introduced into the activated carbon pores. Also, for this reason, the specific surface area was reduced from 1099 to 974 m^2^ g^−1^.

On the other hand, during the process of making the modified adsorbent, due to the use of 650 °C heat and the destruction of some surface bands, there is a slight increase in specific surface area. However, the effect of ammonium persulfate particles on the specific surface area change is greater. As a result, the specific surface area of modified AC is lower than that of AC.

### Infrared spectroscopy study

**Figure 2:**
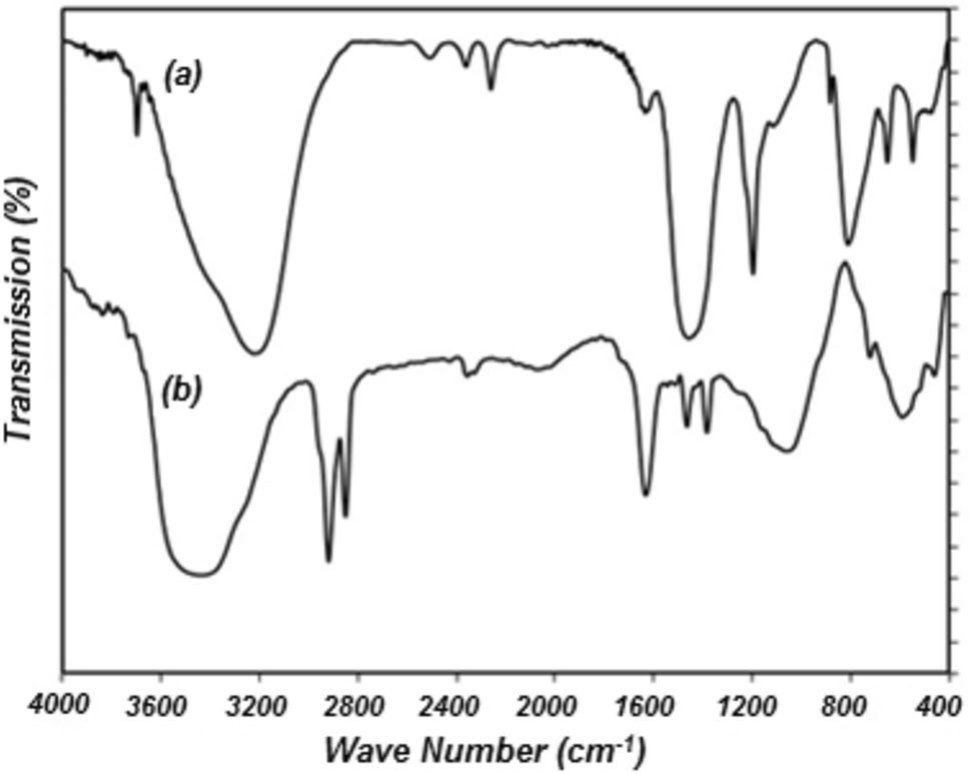
Infrared spectroscopy (**a**) granular AC and (**b**) ammonium persulfate-modified AC.

Infrared spectroscopy was used to investigate the effect of ammonium persulfate on granular AC. The FTIR spectra of granular AC and modified AC are shown in Fig. 2. In the modification process of AC, its surface is oxidized. Therefore, in infrared spectroscopy, the peaks of the oxygen groups already present on the surface increase. For example, the OH group in carboxylic acid structures (in the range of 3500 cm^−1^) and the C–O–C group in the ether structure (in the range of 1200 cm^−1^) are among these.

Asymmetric and symmetric stretching vibrations of the methyl group (-CH_3_-) are also observed at wavelengths of 1379 cm^−1^ and 1469 cm^−1^. The peak created in 1633 cm^−1^ belongs to the carboxylic acid group (C = O). The peaks formed at 2851 and 2922 cm^−1^ are related to the C-H bond. The peak in the range of 3500 cm^−1^ is related to the O–H bond. However, the peak belonging to the OH-group in the phenolic structure (1462 cm^−1^) becomes two peaks, 1379 and 1462 cm^−1^. After increasing the oxidation time, the phenolic groups become lactone groups ^30,31^.

### Effect of pH

**Figure 3:**
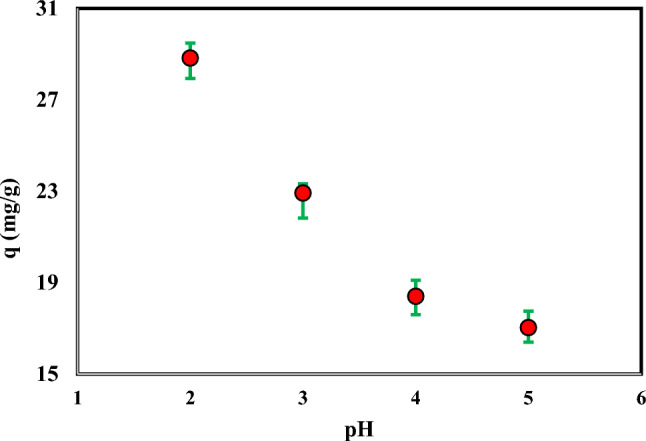
Effect of pH on the adsorption of Cr(VI) by ammonium persulfate-modified AC (Agitation time 3 h, Temperature: 298.15 K, Speed: 150 rpm, Cr: 100 mg L^-1^, Adsorbent mass: 0.03 g, and Solution volume: 10 mL).

The influence of initial pH on the adsorption of Cr(VI) ions was investigated in the range of 2–5. The pH of the solutions was adjusted by a solution of 0.1 mol L^−1^ HCl and NaOH. The experimental conditions such as adsorbent dosage, shaker speed, initial Cr concentration and time were determined from other studies. The acquired results are displayed in Fig. 3. The results showed that with increasing pH, the adsorption of Cr(VI) decreased. Therefore, the highest Cr(VI) adsorption occurred at a pH of 2.

In aqueous solutions, Cr(VI) is present as CrO_4_^2−^, Cr_2_O_7_^2−^,and HCrO^4−^ species. In acidic solutions with concentrations greater than 500 mg L^−1^, Cr_2_O_7_^2−^ is the predominant species. While CrO_4_^2−^ and HCrO^4−^ species are predominant species at pHs 2 to 6 and concentrations less than 500 mg L^−1^^32,33^.

Therefore, according to the concentration and pH of the solution, the species in this range are CrO_4_^2-^ and HCrO^4-^. Possible ion exchange reactions between the AC surface and different species of Cr(VI) are the following equilibrium reactions^34^. The C is the symbol of AC in all of the following reactions:

4 $$\underline {C} OH_{2}^{ + } + HCrO_{4}^{ - } \rightleftarrows \underline {C} HCrO_{4} + H_{2} O$$

5 $$\underline {C} OH + HCrO_{4}^{ - } \rightleftarrows \underline {C} HCrO_{4} + OH^{ - }$$

6 $$\underline {C} OH_{2}^{ + } + CrO_{4}^{2 - } \rightleftarrows \underline {C} CrO_{4}^{2 - } + H_{2} O$$

7 $$\underline {C} OH + CrO_{4}^{2 - } \rightleftarrows \underline {C} CrO_{4}^{2 - } + OH^{ - }$$

Adsorption reactions that can take place between new functional groups on the AC surface and different species of Cr(VI) are as follows^17^:

8 $$\underline {C} - C = O_{\left( s \right)} + HCrO_{4}^{ - } + H_{{\left( {aq} \right)}}^{ + } \to \underline {C} - C = OH^{ + } \ldots HCrO_{4\left( s \right)}^{ - }$$

9 $$\underline {C} - COOH_{\left( s \right)} + CrO_{4}^{2 - } + H_{{\left( {aq} \right)}}^{ + } \to [\underline {C} - COOH_{2}^{ + } ]_{2} ....CrO_{4\left( s \right)}^{2 - }$$

10 $$\underline {C} - C = O_{\left( s \right)} + HCrO_{4}^{ - } + H_{{\left( {aq} \right)}}^{ + } \to \underline {C} - C = OH^{ + } ....HCrO_{4\left( s \right)}^{ - }$$

11 $$\underline {C} - C = O_{\left( s \right)} + CrO_{4}^{2 - } + H_{{\left( {aq} \right)}}^{ + } \to [\underline {C} - C = OH^{ + } ]_{2} ....CrO_{4\left( s \right)}^{2 - }$$

12 $$\underline {C} - OH_{\left( s \right)} + HCrO_{4}^{ - } + H_{{\left( {aq} \right)}}^{ + } \to \underline {C} - OH_{2}^{ + } ....HCrO_{4\left( s \right)}^{ - }$$

13 $$\underline {C} - OH_{\left( s \right)} + CrO_{4}^{2 - } + H_{{\left( {aq} \right)}}^{ + } \to [\underline {C} - OH_{2}^{ + } ]_{2} ....CrO_{4\left( s \right)}^{2 - }$$

The Cr(VI) removal process combines ion exchange and adsorption. Due to the equilibrium reactions of ion exchange and high adsorption, it can be concluded that the more acidic the environment, the higher the adsorption of Cr(VI) species. The obtained results are in good agreement with those above Therefore, other tests were performed at pH = 2.

### Kinetic study of adsorption

**Figure 4:**
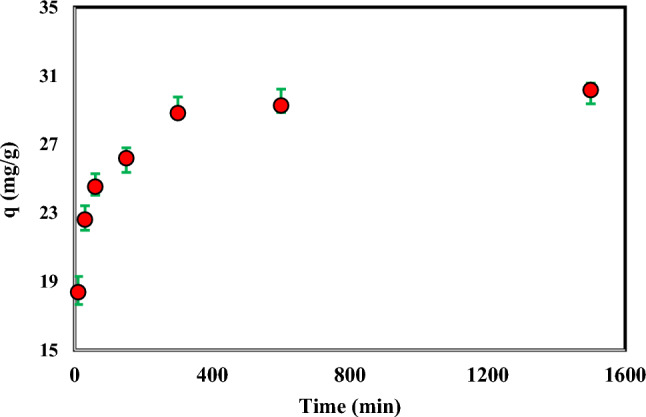
Effect of time on the adsorption of Cr(VI) by ammonium persulfate-modified AC (Temperature: 298.15 K, Speed: 150 rpm, Cr: 100 mg L^-1^, Adsorbent mass: 0.03 g, and Solution volume: 10 mL).

The impact of time on the adsorption of Cr(VI) ions on the modified AC adsorbent is depicted in Fig. 4. To investigate the adsorption kinetics, Cr(VI) adsorption experiments were performed for a long time (1500 min). Pseudo-first-order kinetics models and pseudo-second-order kinetics models have been investigated to determine the degree of the adsorption kinetics equation. The phenomenon of Cr(VI) adsorption displays a gradual increase over time, followed by a subsequent decrease in the adsorption rate due to the progressive occupation of the active sites on the adsorbent until a state of equilibrium is attained. The findings indicate that a substantial portion, exceeding 90%, of the adsorption process occurs within the initial 300-min timeframe. Therefore, other tests were performed in 300 min.

**Figure 5:**
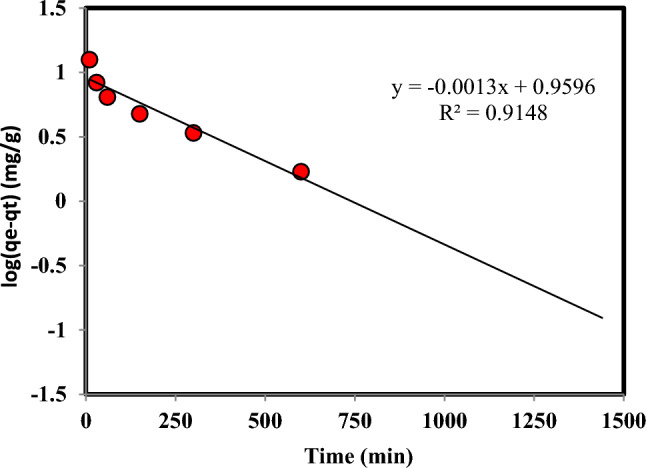
The pseudo-first-order kinetic model for the adsorption of Cr(VI) on persulfate-modified AC (Temperature: 298.15 K, Speed: 150 rpm, Cr: 100 mg L^-1^, Adsorbent mass: 0.03 g, and Solution volume: 10 mL).

**Figure 6:**
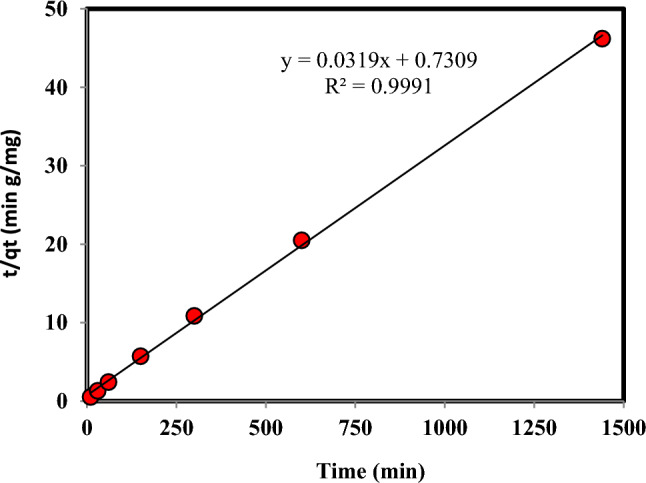
The second-order kinetic model for the adsorption of Cr(VI) on persulfate-modified AC (Temperature: 298.15 K, Speed: 150 rpm, Cr: 100 mg L^-1^, Adsorbent mass: 0.03 g, and Solution volume: 10 mL).

The kinetic information regarding the adsorption of Cr(VI) by the adsorbent was examined using the following equations, which employed the pseudo-first-order and pseudo-second-order approaches^35–37^:

14 $${\text{q}}_{{\text{t}}} = {\text{ q}}_{{\text{e}}} { }\left( {1 - {\text{e}}^{{ - {\text{k}}_{1} {\text{t}}}} } \right)$$

15 $${\text{q}}_{{\text{t}}} { = }\frac{{{\text{q}}_{{\text{e}}}^{2} {\text{k}}_{2} {\text{t}}}}{{1 + {\text{q}}_{{\text{e}}} {\text{k}}_{2} {\text{t}}}}$$

where the adsorption capacity at time t and the adsorption capacity at equilibrium time, as well as the kinetic constant, are represented by q_t_, q_e_, and k, respectively. The graphs illustrating the pseudo-first-order and pseudo-second-order kinetic models can be observed in Figs. 5 and 6, respectively. Also, Table 2 presents the results and percentage of compliance with these models. The results show that the correlation of Cr(VI) adsorption kinetics with the pseudo-second-order model is much higher than the pseudo-first-order model.

**Table 2:** The parameters of the pseudo-first-order and pseudo-second-order kinetic model of Cr(VI) adsorption on ammonium persulfate-modified AC (Temperature: 298.15 K, Speed: 150 rpm, Cr: 100 mg L^−1^, Adsorbent mass: 0.03 g, and Solution volume: 10 mL).

pseudo-second-order			pseudo-first-order		
R^2^	q_e_ (mg.g^−1^)	K_2_ (g.mg^−1^.min)	R^2^	q_e_ (mg.g^−1^)	K_1_ (min^−1^)
0.999	31.35	0.0014	0.915	9.11	0.003

### Thermodynamic study of adsorption

**Figure 7:**
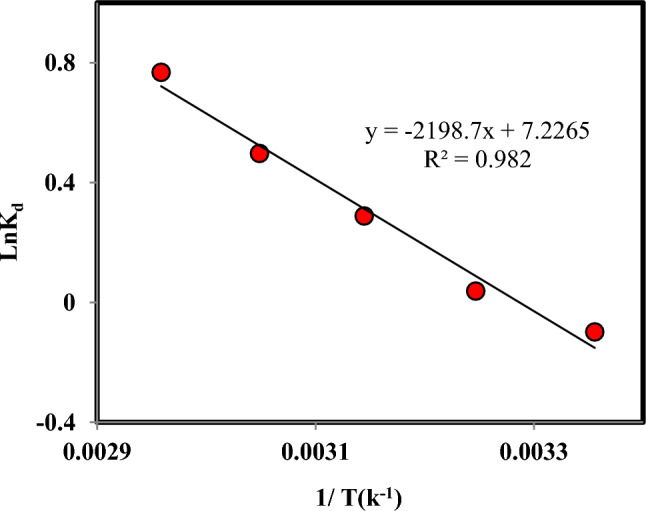
The logarithm variations of the distribution coefficient versus the 1/T for Cr(VI) adsorption on persulfate-modified AC (Agitation time 5 h, Speed: 150 rpm, Cr: 100 mg L^-1^, Adsorbent mass: 0.03 g, and Solution volume: 10 mL).

To determine the thermodynamics of Cr(VI) adsorption with the prepared adsorbent, the values of enthalpy change (ΔH^o^), entropy change (ΔS^o^), and Gibbs free energy change (ΔG^o^) were obtained. The experiments were performed at temperatures of 298.15 to 338.15 K. The Van’t Hoff plots are shown in Fig. 7, and the thermodynamic parameters are obtained from the following Eqs.^38–40^:

16 $$\text{ln}{K}_{d}=-\frac{\Delta \text{H}}{\text{RT}}+ \frac{\Delta \text{S}}{\text{R}}$$

17 $${\Delta \text{G}}^{o}={ \Delta \text{H}}^{o}-{\text{T }\Delta \text{S}}^{o}$$

where R, T, and K_d_ are the universal gas constant (kJmol^-1^K^-1^), the absolute temperature (K), and the distribution coefficient (cm^3^/g), respectively^41^. The ΔH^o^ and ΔS^o^ values are determined by drawing ln K_d_ vs. 1/T. The results are displayed in Table 3.

**Table 3:** Thermodynamic parameters of Cr(VI) adsorption on persulfate-modified AC (Agitation time 5 h, Speed: 150 rpm, Cr: 100 mg L^−1^, Adsorbent: 0.03 g, and Solution volume: 10 mL).

ΔH^◦^ (kJ.mol^−1^)	ΔS^◦^ (kJ^−1^.mol^−1^.K^−1^)	ΔG^◦^ (kJ.mol^−1^)					R^2^
		298	308	318	328	338	
18.28	0.06	− 2.63	− 3.23	− 4.36	− 5.54	− 6.58	0.982

The value of ΔH^o^ was calculated at 18.28 kJ mol^-1^. The positive value of ΔH^o^ shows that Cr(VI) adsorption is endothermic, and the adsorption will enhance with increasing temperature. The value of ΔS^o^ is 0.06 J K^−1^mol^−1^, which shows that the Cr(VI) adsorption is random ^42^. The ΔG^o^ values in this temperature range are negative, and the adsorption reaction is spontaneous. The value of ΔG^o^ is in the range of -20 to 0 kJ; it can be said that Cr(VI) adsorption on persulfate-modified AC is physical adsorption.

### Effect of ionic strength

**Figure 8:**
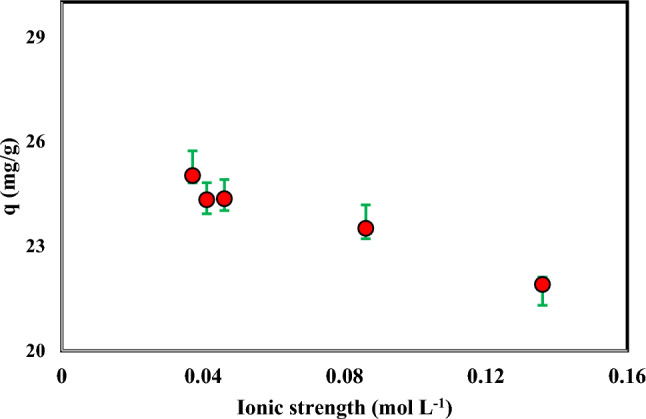
Effect of ionic strength on the adsorption of Cr(VI) by ammonium persulfate-modified AC (Agitation time 5 h, Temperature: 298.15 K, Speed: 150 rpm, Cr: 100 mg L^-1^, NaCl: 0.0001–0.1 mol L^-1^, Adsorbent mass: 0.03 g, and Solution volume: 10 mL).

The impact of ionic strength on the adsorption capacity of Cr(VI) was investigated. The results are shown in Fig. 8. The results showed that the adsorption of the Cr(VI) ion on ammonium persulfate-modified AC did not change significantly with increasing ionic strength. As a result, sodium and increased ionic strength have almost no effect on the adsorption of Cr(VI) ions.

### Effect of initial ion concentration (Isotherm Study)

The effect of the initial concentration of Cr(VI) in the feed solution in the range of 5 to 600 mg L^-1^ on the adsorption was investigated. The Freundlich and Langmuir adsorption isdotherm models have been used to investigate the adsorption process. The mathematical expressions of the Freundlich and Langmuir adsorption isotherm models are expressed in the following Eqs.^43–45^:

18 $${\text{q}}_{e} = {\text{K}}_{F} {\text{C}}_{e}^{\frac{1}{n}}$$

19 $${\text{q}}_{{\text{e}}} = \frac{{{\text{q}}_{{\text{L}}} {\text{K}}_{{\text{L}}} {\text{C}}_{{\text{e}}} }}{{1 + {\text{K}}_{{\text{L}}} {\text{C}}_{{\text{e}}} }}$$

where the q_e_ and q_L_ represent the adsorption capacity at equilibrium and the maximum adsorption capacity according to the Langmuir isotherm, respectively. K_F_ and K_L_ are the model’s constants. The extent of deviation from linearity in adsorption is indicated by n.

**Figure 9:**
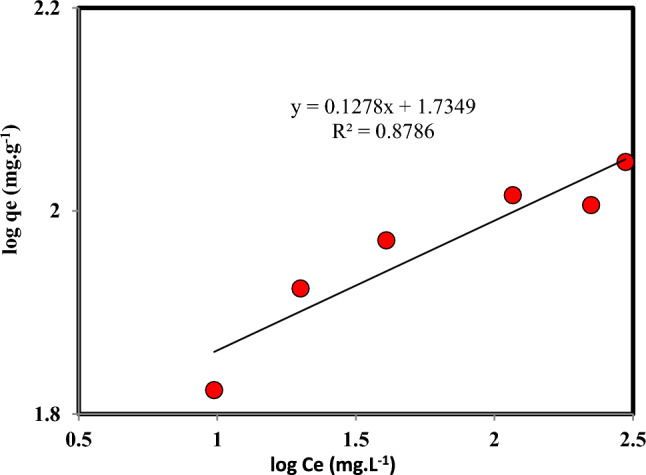
Freundlich adsorption isotherm plot for the adsorption of Cr(VI) on persulfate-modified AC (Agitation time 5 h, Temperature: 298.15 K, Speed: 150 rpm, Cr: 5–600 mg L^-1^, Adsorbent mass: 0.03 g, and Solution volume: 10 mL).

**Figure 10:**
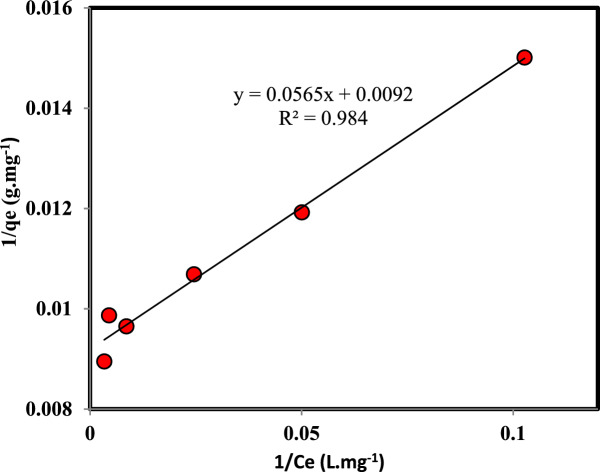
Langmuir adsorption isotherm plot for adsorption of Cr(VI) on persulfate-modified AC (Agitation time 5 h, Temperature: 298.15 K, Speed: 150 rpm, Cr: 5–600 mg L^-1^, Adsorbent mass: 0.03 g, and Solution volume: 10 mL).

The experimental results of Cr(VI) adsorption based on the Freundlich and Langmuir linear models of adsorption are shown in Figs. 9 and 10. The results of the equilibrium data modeling indicated that the data fitting using the Langmuir model (R^2^ = 0.98) is superior to that of the Freundlich model (R^2^ = 0.88). So, adsorption will be monolayer, and the adsorbent surface is homogeneous.

**Figure 11:**
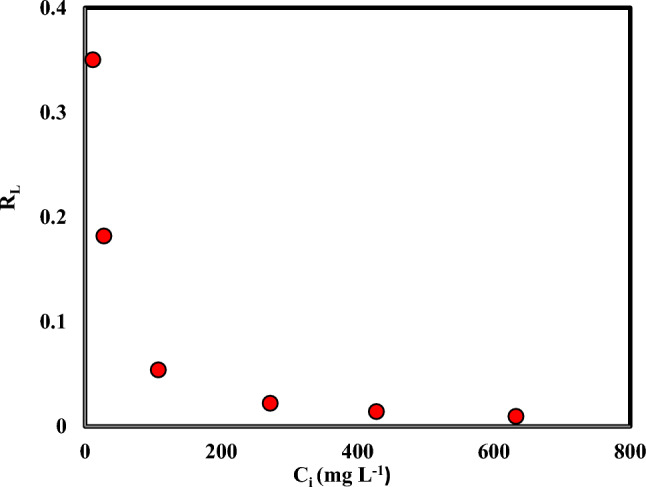
Variations of the dimensionless equilibrium parameter (RL) versus the initial concentration (Agitation time 5 h, Temperature: 298.15 K, Speed: 150 rpm, Cr: 5–600 mg L^-1^, Adsorbent mass: 0.03 g, and Solution volume: 10 mL).

Variations of the dimensionless equilibrium parameter (R_L_) versus the initial concentration are shown in Fig. 11. The R_L_ values between 0 and 1 indicate the desirability of the adsorption process. With the increase in ion concentration, the value of R_L_ will be smaller, and the adsorption process will be more favorable. The findings presented in Table 4 showed that 108.69 mg g^-1^ is the maximal Cr(VI) adsorption capacity on persulfate-modified AC adsorbent.

**Table 4:** The Langmuir and Freundlich isotherm parameters for Cr(VI) adsorption with persulfate-modified AC (Agitation time 5 h, Temperature: 298.15 K, Speed: 150 rpm, Cr: 5–600 mg L^−1^, Adsorbent mass: 0.03 g, and Solution volume: 10 mL).

Freundlich			Langmuir		
R^2^	K_f_ (mg.g^−1^.mg^−m^.L^m^)	n	R^2^	K_L_ (L.mg^−1^)	q_max_ (mg.g^−1^)
0.8786	31.73	0.1368	0.984	0.1628	108.69

Table 5 compares the adsorption of Cr(VI) by the persulfate-modified AC adsorbent and other types of AC adsorbents. This comparison shows that the adsorption properties are intensified with the modification of AC by persulfate. So, the ability to absorb Cr(VI) by this adsorbent is more than twice that of other AC adsorbents. High adsorption capacity and simple preparation method showed that ammonium persulfate modified-activated carbon adsorbent can be a promising Cr adsorbent.

**Table 5:** Comparison of Cr(VI) adsorption by persulfate-modified AC adsorbent with other types of AC adsorbents.

Adsorbent type	Modifier	Operating conditions	Maximum adsorption capacity (mg g^−1^)	Maximum adsorption (%)	Ref
Granulated activated carbon (GAC-S)	nitric acid, ammonium persulphate and hydrogen peroxide	Cr (VI) = 20–1000 mg/L, ambient temperature	30	–	^46^
Activated carbon prepared from coconut tree sawdust	H_2_SO_4_	Dosage = 50–750 mg, V_solution_ = 50 mL, Time = 180 min, pH = 3, Cr (VI) = 5–20 mg/L, shaker = 150 rpm, ambient temperature	3.46	–	^47^
Commercial coconut activated carbon	HNO_3_ and NaOH	Dosage = 0.2 g, V_solution_ = 100 mL, Cr (VI) = 5–50 mg/L, Temp = 303.15 K	13.88	–	^12^
Activated carbon	Modification of AC with 7 mol/L of HNO_3_ at 90 ^o^C	Dosage = 2 g, V_solution_ = 100 mL, Cr (VI) = 10 mg/L, Temp = 301.15 K	10.929	83	^13^
Activated carbon	HNO_3_	Dosage = 0.2–2.5 g, Time = 180 min, pH = 2–9, Cr (VI) = 25 mg/L, ambient temperature	16.10	99	^18^
Activated carbon (Made from palm bark)	polyethylene amine (228.2 mg/g PEI on AC)	Dosage = 0.1 g, V_solution_ = 50 mL, Time = 24 h, pH = 3–4, Cr (VI) = 200 mg/L, shaker = 150 rpm, ambient temperature	20.5	76.64	^14^
Activated carbon	Fe (III)	Dosage = 0.25–5 g/L, Time = 1–500 min, pH = 2–11, Cr (VI) = 3.5–20 mg/L, shaker = 300 rpm, ambient temperature	11.83	100	^15^
Commercial activated carbon	–	Dosage = 6 g/L, Time = 24 h, pH = 6, Cr (VI) = 50 mg/L, shaker = 250 rpm, ambient temperature	52.60	–	^16^
Activated carbon	(NH_4_)_2_S_2_O_8_	Dosage = 0.03 g, V_solution_ = 10 mL Time = 180 min, pH = 2–5, Cr (VI) = 100 mg/L, shaker = 150 rpm, ambient temperature	108.69	90.49	Present study

## Conclusions

In this research, a new adsorbent was used to extract Cr(VI) from aqueous solution. Ammonium persulfate is used to modify granular activated carbon to prepare the new adsorbent. The adsorbent properties were characterized thanks to nitrogen adsorption and desorption isotherm data and infrared spectroscopy. The activated carbon modification with ammonium persulfate resulted in a reduction in both the average pore size and specific surface area. The infrared spectroscopy showed that the oxygen groups' peaks on the surface increased.

Batch experiments were used to investigate the impact of process variables on the adsorption, including the initial pH value of the solution, time, temperature, ionic strength, and initial concentration of the Cr(VI) ion. The results showed that Cr(VI) adsorption decreases with increasing pH, and the highest Cr(VI) adsorption occurred at pH 2. The findings showed that the first 300 min account for more than 90% of the adsorption, and the correlation of adsorption kinetics with the pseudo-second-order model is higher than the pseudo-first-order model. Thermodynamic analyses demonstrated that adsorption is a spontaneous, endothermic reaction. The results showed that the adsorption does not change significantly with increasing ionic strength.

The study of the adsorption isotherm showed that the adsorption process is consistent with the Langmuir model. The maximum adsorption capacity of Cr(VI) onto persulfate-modified activated carbon was obtained at 108.69 mg g^−1^. By comparing the obtained results with those of other adsorbents, it can be argued that activated carbon adsorbent modified with persulfate is an excellent adsorbent for Cr(VI) removal from aqueous solutions.

## Data Availability

Data availability The datasets used and/or analyzed during the current study are available from the corresponding author on reasonable request.

## List of symbols

*Ci*: Initial adsorbate concentration (mg L^−^^1^)
*C*_*f*_: Equilibrium adsorbate concentration (mg L^−^^1^)
*C*_*t*_: Adsorbate concentration at time *t* (mg L^−^^1^)
*V*: Solution volume (mL)
*m*: Adsorbent mass (g)
AC: Activated carbon
*k*_1_: Pseudo-first-order rate constant (min^−^^1^)
*k*_2_: Pseudo-second-order rate constant (g mg^−^^1^ min^−^^1^)
*K*_*d*_: Distribution factor
*K*_*F*_: Freundlich isotherm constant ((mg g^−^^1^)(L mg^−^^1^)^1/n^)
*K*_*L*_: Langmuir isotherm constant (L mg^−^^1^)
*n*: Freundlich intensity constant
*q*_*e*_: Adsorption capacity at equilibrium (mg g^−^^1^)
*q*_*L*_: Langmuir adsorption capacity (mg g^−^^1^)
*q*_*t*_: Adsorption capacity at time *t* (mg g^−^^1^)
*R*: Universal gas constant (kJ mol^−^^1^ K^−^^1^)
*R*^*2*^: Determination coefficient
*t*: Time (min)
*T*: Temperature (K)
Δ*G°*: Gibbs free energy change (kJ mol^−^^1^)
Δ*H°*: Enthalpy change (kJ mol^−^^1^)
Δ*S°*: Entropy change (J mol^−^^1^ K^−^^1^)
